# Molecular memory of *Flavescence dorée* phytoplasma in recovering grapevines

**DOI:** 10.1038/s41438-020-00348-3

**Published:** 2020-08-01

**Authors:** Chiara Pagliarani, Giorgio Gambino, Alessandra Ferrandino, Walter Chitarra, Urska Vrhovsek, Dario Cantu, Sabrina Palmano, Cristina Marzachì, Andrea Schubert

**Affiliations:** 1 Institute for Sustainable Plant Protection, National Research Council (IPSP-CNR), Strada delle Cacce 73, 10135 Turin, Italy; 2 PlantStressLab, Department of Agricultural, Forestry and Food Sciences, University of Turin, Largo Paolo Braccini 2, 10095 Grugliasco, TO Italy; 3 Research Centre for Viticulture and Enology, Council for Agricultural Research and Economics (CREA-VE), Via XXVIII Aprile 26, 31015 Conegliano, TV Italy; 4 Fondazione Edmund Mach, Via Edmund Mach 1, 38010 San Michele all’Adige, TN Italy; 5 Department of Viticulture and Enology, University of California, One Shields Avenue, Davis, CA 95616 USA

**Keywords:** Biotic, Plant physiology

## Abstract

*Flavescence dorée* (FD) is a destructive phytoplasma disease of European grapevines. Spontaneous and cultivar-dependent recovery (REC) may occur in the field in FD-infected vines starting the year following the first symptoms. However, the biological underpinnings of this process are still largely unexplored. In this study, transcriptome sequencing (RNAseq), whole-genome bisulphite sequencing (WGBS) and metabolite analysis were combined to dissect molecular and metabolic changes associated to FD and REC in leaf veins collected in the field from healthy (H), FD and REC plants of the highly susceptible *Vitis vinifera* ‘Barbera’. Genes involved in flavonoid biosynthesis, carbohydrate metabolism and stress responses were overexpressed in FD conditions, whereas transcripts linked to hormone and stilbene metabolisms were upregulated in REC vines. Accumulation patterns of abscisic acid and stilbenoid compounds analysed in the same samples confirmed the RNAseq data. In recovery conditions, we also observed the persistence of some FD-induced expression changes concerning inhibition of photosynthetic processes and stress responses. Several differentially expressed genes tied to those pathways also underwent post-transcriptional regulation by microRNAs, as outlined by merging our transcriptomic data set with a previously conducted smallRNAseq analysis. Investigations by WGBS analysis also revealed different DNA methylation marks between REC and H leaves, occurring within the promoters of genes tied to photosynthesis and secondary metabolism. The results allowed us to advance the existence of a “molecular memory” of FDp infection, involving alterations in the DNA methylation status of REC plants potentially related to transcriptional reprogramming events, in turn triggering changes in hormonal and secondary metabolite profiles.

## Introduction

Grapevine (*Vitis vinifera* L.) is the main fruit crop in temperate climates and is cultivated in all continents for fresh and dry fruit consumption and for wine making. A major problem affecting this species is its susceptibility to diseases and pests. Nowadays, sustainable crop protection is one of the most urgent challenges in viticulture. The discovery of plant traits controlling susceptibility to diseases is a key issue to proceed in this direction.

In grapevine, phytoplasma infection induces a complex of symptoms, and the related diseases are commonly referred to as grapevine yellows (GY)^1^; the most economically important GY diseases in Europe are *Bois Noir* (BN) and *Flavescence dorée* (FD). The BN is due to infection by ‘*Candidatus* Phytoplasma solani’ (16SrXII-A, stolbur group), and is transmitted by the polyphagous leafhopper *Hyalesthes obsoletus*^2^. The FD disease emerged relatively recently; it was first detected in France in the 1960s, then it quickly spread to most European grape-growing areas^3^. Its causal agent is a phytoplasma (FDp) belonging to the elm-yellows group (16SrV-C and -D) and transmitted by the grape-specific leafhopper *Scaphoideus titanus*^4^. FD causes heavy damage to vineyards in Europe, as only indirect methods, such as control of the vector population with insecticide treatments, uprooting of infected plants, and replanting of healthy (heat-treated during propagation) plants, are available for containing the infection. However, these countermeasures pose a heavy burden in terms of environmental and economical sustainability. No resistance to FDp has been detected in *V. vinifera* yet, although different levels of tolerance do exist among cultivars^5–7^.

A peculiar and still little understood aspect of grapevine phytoplasma diseases is the process of spontaneous recovery, whereby in a new vegetation year infected plants become symptomless and negative to diagnostic tests^8^. Recovery is observed for both FDp- and BNp-infected plants^8–11^. Events of FD recovery have been monitored in different *V. vinifera* varieties, across diverse geographical areas^8^, and field observations attested that recovery rates significantly change according to environmental conditions and host genotype^5,8,12,13^. Mathematical projections, achieved through statistical analysis of space-time dynamic patterns of FD recovery over the years, outlined that, in the susceptible cultivar Barbera, the percentages of recovery average from 25 to 35% over 4–5 years following the original infection^13^. In another multi-year survey, conducted on different cultivars, Morone et al.^8^ found a mean recovery rate around 65–70%. Finally, in a 2-year observation trial performed on two cultivars with different susceptibility to FDp, other authors reported an annual mean recovery rate of 56% in the highly susceptible ‘Barbera’ and of 21% in the less susceptible ‘Nebbiolo’^14^. These data, coupled with quantitative molecular diagnostic analyses showing higher FDp loads in ‘Barbera’ than ‘Nebbiolo’^15^, suggest that genotypes experiencing more severe infections are more primed to recover from the disease. Hence, understanding the biological bases affecting establishment, rates and maintenance of recovery could lead to increase recovery occurrence and, consequently, to reduce symptoms and disease severity within a vineyard.

The different degrees of FD tolerance observed in the field may be due to either native immunity-like mechanisms reducing insect vector attractiveness, phytoplasma spread or multiplication within the plant, or induced immunity-like events triggering recovery from the disease. Both processes should be explored in order to allow for breeding new disease-resistant genotypes and develop new strategies of sustainable disease management. Over the last years, changes induced by FD were explored at the metabolic, molecular^7,16,17^ and physiological^14^ levels. These approaches, however, have never been extended to the whole grapevine transcriptome in the case of FDp, whereas transcriptomic analyses were performed in plants affected by BNp^11,18^. Molecular markers involved in induced immunity-like recovery are more difficult to characterise using differential transcript or metabolite analysis, as recovered plants can be identified only when the remission process is completed, that could happen starting the year after the first infection. Recovered vines may remain symptomless for years, although preliminary data indicated that they can be infected again by the same phytoplasma^19^. Thus, analyses have been focused on stably (more than 2-year-old) recovered plants, surmising on the persistence of marks induced by FD and/or recovery in the plant tissues even after the disappearance of the phytoplasma. Following this approach, selected metabolic pathways were shown to be modified in plants stably recovered from FDp^7,12,16^ and BNp^9,11^ compared to healthy ones. These outcomes implicitly confirm the hypothesis of persistence of recovery-induced molecular modifications. However, no such study has been performed at the ‘omic’ level up to now.

As recovery from FD seems to induce persistent molecular changes, epigenetic-based mechanisms are obvious targets to search for recovery-dependent molecular processes. Experimental evidence has revealed that gene transcriptional reprogramming may be controlled by epigenetic modifications in turn triggered by various pathogenic challenges^20^. Nevertheless, changes in DNA epigenetic landscapes underpinning the interplay between grapevine and stress factors are still poorly characterised^21^, and according to our knowledge, they have never been investigated in correlation with spontaneous recovery from phytoplasma disease.

Secondary metabolites, such as phytoalexins, are typically activated upon pathogen attack in plants. Stilbenes are the major known phytoalexins in grapevine, they strongly increase under biotic stress^22^ and they effectively hinder the spread of fungal pathogens in vitro and in vivo^23^. Importance of these molecules in grapevine is also underlined by the presence of a wide stilbene synthase (*VvSTS*) gene family^24^ and of numerous stilbene compounds, including *trans*-resveratrol, *trans*-piceid^25^, piceatannol^26^, pterostilbenes and polymerised stilbenes (viniferins)^27^. Given these assumptions, the involvement of stilbenes in the recovery following FDp infection can be envisaged. In addition, the perturbation of hormone signalling pathways in plants affected by and recovering from phytoplasma diseases deserves attention^28,29^. Indeed, specific hormonal signals are crucial in the induction of recovery phenomena^30^ and in the regulation of stilbene biosynthesis^31^.

In this study we used different approaches to identify molecular processes underlying recovery from FD in grapevine. To this aim, we quantitatively compared the leaf transcriptomes and levels of key secondary metabolites and hormones of stably recovered (REC) vines with those of healthy (H) and infected (FD) plants. Then, in search for molecular evidence supporting FDp infection/recovery-induced reprogramming of defence responses, we analysed DNA methylation profiles of REC and H plants.

## Results

### Overview of the main molecular modifications induced in FD and REC samples

We first addressed our survey to investigate the whole transcriptome reprogramming events occurring in FDp-infected and stably recovered (2-year-old REC) leaf veins making comparisons with the transcriptome of samples collected from H plants. This allowed us to provide a comprehensive overview of changes in the main functional gene categories associated with either disease or recovery condition.

**Fig. 1:**
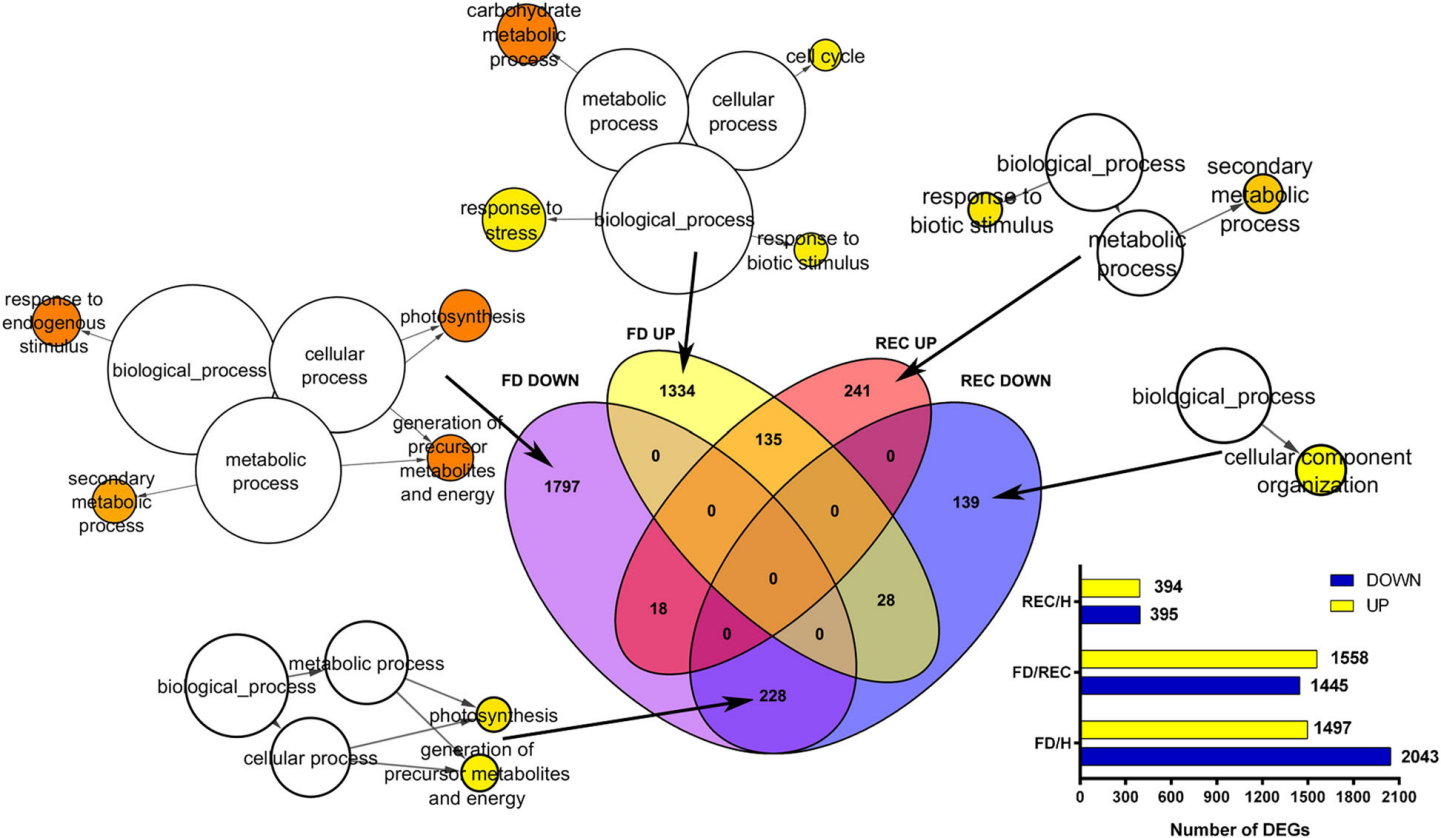
Transcriptome analysis of FDp-infected (FD) and recovered (REC) samples. The Venn diagrams in the middle of the figure report the number of either exclusive or common upregulated and downregulated genes resulting by merging RNAseq data of the FD vs healthy (H) comparison with those of the REC vs H comparison. When significant, the enrichment of specific GO biological process terms is shown for each group of DEGs from the Venn charts. The significant enriched GO biological functional categories were retrieved using Cytoscape with the BINGO plug-in and listed according to their enrichment *P*‐value (*P* < 0.05). The total numbers of up and down differentially expressed genes (DEG) are visualised for each RNAseq comparison in the bar charts displayed right in the bottom

RNAseq analysis produced an average of 47 million fragments per sample that were quality filtered and aligned to the PN40024 reference genome (IGGP 12X v. 16 assembly) with a mean mapping rate of 91% (Supplementary Table S1). Out of the 29,970 annotated genes, 6689 (22.3%) were significantly differentially expressed (DEGs) in at least one of the three comparisons, namely FD vs H, REC vs H and FD vs REC (*P*-value adjusted with Benjamin–Hochberg ≤ 0.5%). A fold-change (FC) cut-off threshold was applied to reduce data representation and only the 4707 DEGs whose expression level was |log2FC| ≥ 1 were further analysed (Supplementary Table S2). Elaboration of sequencing data highlighted a much higher number of expression changes in FD vs H (3540 DEGs in total, 1497 upregulated and 2043 downregulated) and FD vs REC (3003 DEGs in total, 1558 upregulated and 1445 downregulated) than REC vs H comparison (789 DEGs in total, 394 upregulated and 395 downregulated) (Fig. 1; Supplementary Table S2).

In total, 1334 and 1797 transcripts were respectively upregulated and downregulated by FD compared to H (Fig. 1; Supplementary Table S3). Carbohydrate metabolic processes, response to stress and response to biotic stimulus and cell cycle were the categories significantly over-represented among FD vs H upregulated DEGs (Fig. 1; Supplementary Table S3). The Carbohydrate metabolic process class featured upregulation of several genes encoding vacuolar invertases and sucrose synthases, consistently with FD-dependent accumulation of soluble sugars^32^. Further highly expressed DEGs belonging to this functional group encoded ADP-glucose pyrophosphorylase (AGPase) and starch synthase, whose activity was shown to increase significantly following FDp infection, contributing to starch accumulation in infected tissues^32^. On the contrary, photosynthesis, generation of precursor metabolites and energy, response to endogenous stimulus (i.e. hormone signalling associated genes), and secondary metabolic processes, were the enriched gene categories among FD-downregulated DEGs (Fig. 1, Supplementary Table S3). The downregulation of genes included in the first two classes is a well-known hallmark of FD infection^14^. The enriched secondary metabolic process class encompassed genes involved in terpenoid and phenylpropanoid biosynthesis. However, the transcription of the main transcripts of the flavonoid biosynthetic pathway increased in presence of the pathogen. Several transcription factors associated to activation of anthocyanin and proanthocyanidin biosynthesis, such as MybA1^33^ and MYBPA1^34^, as well as the main anthocyanin biosynthetic genes (i.e. *UFGT*, *AOMT*, *3AT*, *GST4*), were exclusively upregulated in FD veins (Table S3), in accordance with previous observations^7^. Analysis of the FD transcriptome thus revealed gene expression changes linked to severe metabolic limitations, in agreement with physiological alterations previously described in ‘Barbera’ FDp-infected plants^14^.

The comparison between REC and H RNAseq data only evidenced 139 downregulated DEGs and 241 upregulated DEGs (Fig. 1). This implied that, as for the phenotypes, also transcriptomes of samples from stably recovered and healthy plants were highly similar. Within the group of DEGs exclusively upregulated by REC, the main enriched functional categories were Secondary metabolic processes and Response to biotic stimulus (Fig. 1), in agreement with the activation of several transcripts encoding stilbene synthases (Supplementary Table S3). Although corresponding to a relatively low number of DEGs, these findings suggested that FDp infection could act as priming factor inducing long-lasting effects in REC plants. Thus, compared to H, REC vines maintained transcriptomic differences even after disappearance of FD infection and symptoms. The FD vs REC comparison was largely dominated by transcriptional changes already observed in the FD vs H comparison, confirming that REC and H samples were similar at transcriptome level. Consistently, carbohydrate metabolism was the most enriched category among FD vs REC upregulated transcripts (Supplementary Fig. S1A), whereas photosynthesis and secondary metabolic processes were those showing higher enrichment among FD vs REC downregulated genes (Supplementary Fig. S1B).

Further information was gained by the analysis of genes regulated by both conditions (FD and REC) compared to H. Only a few of them were divergently regulated by FD and REC: 28 in common between FD upregulated and REC downregulated and 18 in common between REC upregulated and FD downregulated (Fig. 1; Supplementary Table S3). This indicated that reversed expression of genes upregulated or downregulated during FDp infection was not the main hallmark of recovery, although part of these transcripts could encode proteins involved in the recovery process. DEGs sharing the same expression pattern in FD and REC were limited, but, interestingly, the 228 DEGs downregulated by both FD and REC included several transcripts involved in photosynthesis and energy metabolism (Fig. 1; Supplementary Table S3). In parallel, among the 135 DEGs commonly upregulated in FD and REC, the majority of them encoded proteins belonging to carbohydrate metabolism and stress defence (Supplementary Table S3). These results further supported the persistence of some FD-induced expression alterations during and after the recovery process that could represent either metabolic remnants of FD infection or long-lasting FD-induced molecular reprogramming mechanisms.

As transcript modulation can be influenced by several factors, including post-transcriptional regulation by microRNAs (miRNAs), transcriptomic data were put in correlation with outcomes from a previously published small RNA analysis^35^ conducted on the same plant material. Among the 497 target genes of the miRNAs already validated in grapevine (miRVIT database, http://mirvit.ipsp.cnr.it/), 163 transcripts underwent significant expression differences in at least one of the three comparisons (FD vs H, REC vs H and FD vs REC), and overall showed divergent expression profiles than those of related miRNAs (Supplementary Table S4). Interestingly, this group of common transcripts were enriched for functional categories linked to photosynthesis, response to endogenous stimulus and cell development (Supplementary Fig. S2), revealing that multiple molecular regulatory cascades act in a coordinated manner to control the plant response upon FD infection and recovery.

### Focus on genes specifically influenced by either FD or REC

Expression profiles of a group of transcripts among those mostly activated in either FD (anthocyanin- and sugar-related transcripts) or REC samples (stilbene synthase-encoding genes) or repressed in FD (hormone signalling) were further analysed by real-time PCR assay (RT-qPCR). This survey was performed to validate RNAseq results (Supplementary Fig. S3) and to explore more in depth the molecular changes either regulating specific defence responses to the pathogen or underlying physiological mechanisms leading to recovery.

**Fig. 2:**
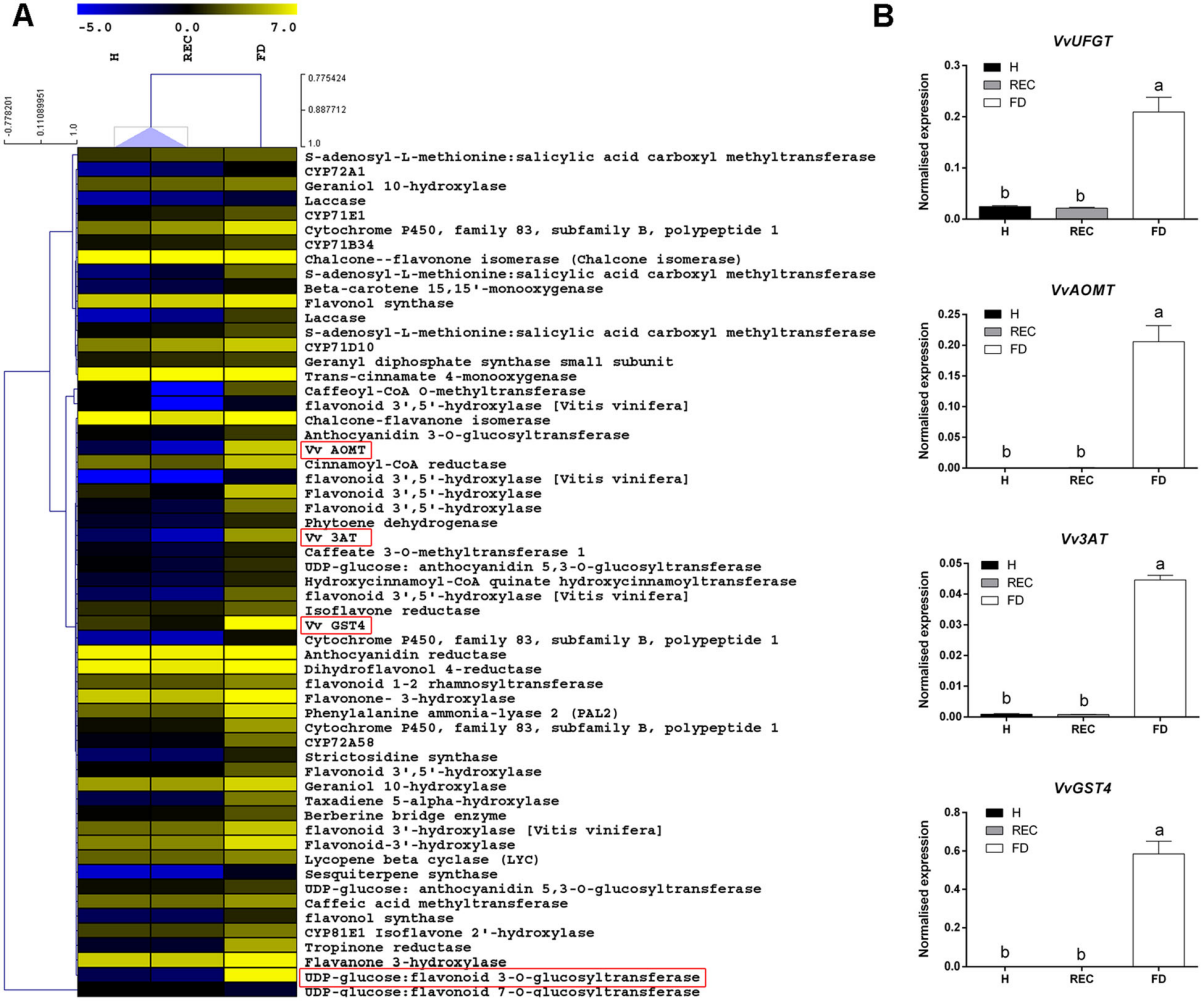
FD-dependent transcriptional reprogramming of secondary metabolic processes. **a** Heat map of differentially expressed transcripts belonging to secondary metabolism, with focus on flavonoid biosynthetic genes. **b** RT-qPCR expression profiles of genes encoding the main anthocyanin biosynthetic enzymes *Vv*UFGT (VIT_16s0039g02230), *Vv*AOMT (VIT_01s0010g03510), *Vv*3AT (VIT_03s0017g00870) and *Vv*GST4 (VIT_04s0079g00690) analysed in leaf veins taken from healthy (H), recovered (REC) or FDp-infected (FD) ‘Barbera’ plants. Ubiquitin (*VvUBI*) and actin (*VvACT1*) genes were both used as endogenous controls for the normalisation of transcriptional levels. Lower case letters denote statistical differences when significant, as determined by Tukey’s HSD test (*P* < 0.05). Bars represent standard error of the mean (*n* = 5)

The FDp infection induced overexpression of flavonoid biosynthetic genes, which formed a well-separated group from REC and H samples, as highlighted by clustering analysis (Fig. 2a).

This was the case of *VvUFGT* (VIT_16s0039g02230) and other genes involved in the final steps of the anthocyanin biosynthetic pathway, all strongly activated in FDp-infected veins (Fig. 2b). Accordingly, transcripts encoding the anthocyanin methyltransferase *VvAOMT*^36^ (VIT_01s0010g03510), among the most expressed within the FD transcriptome data set, were almost undetectable in REC and H samples (Fig. 2b). Similar expression profiles were obtained for the acyltransferase *Vv3AT* (VIT_03s0017g00870) and the glutathione S transferase *VvGST4* (VIT_04s0079g00690) encoding genes, respectively, involved in anthocyanin acylation^37^ and anthocyanin transport^38^ (Fig. 2b).

**Fig. 3:**
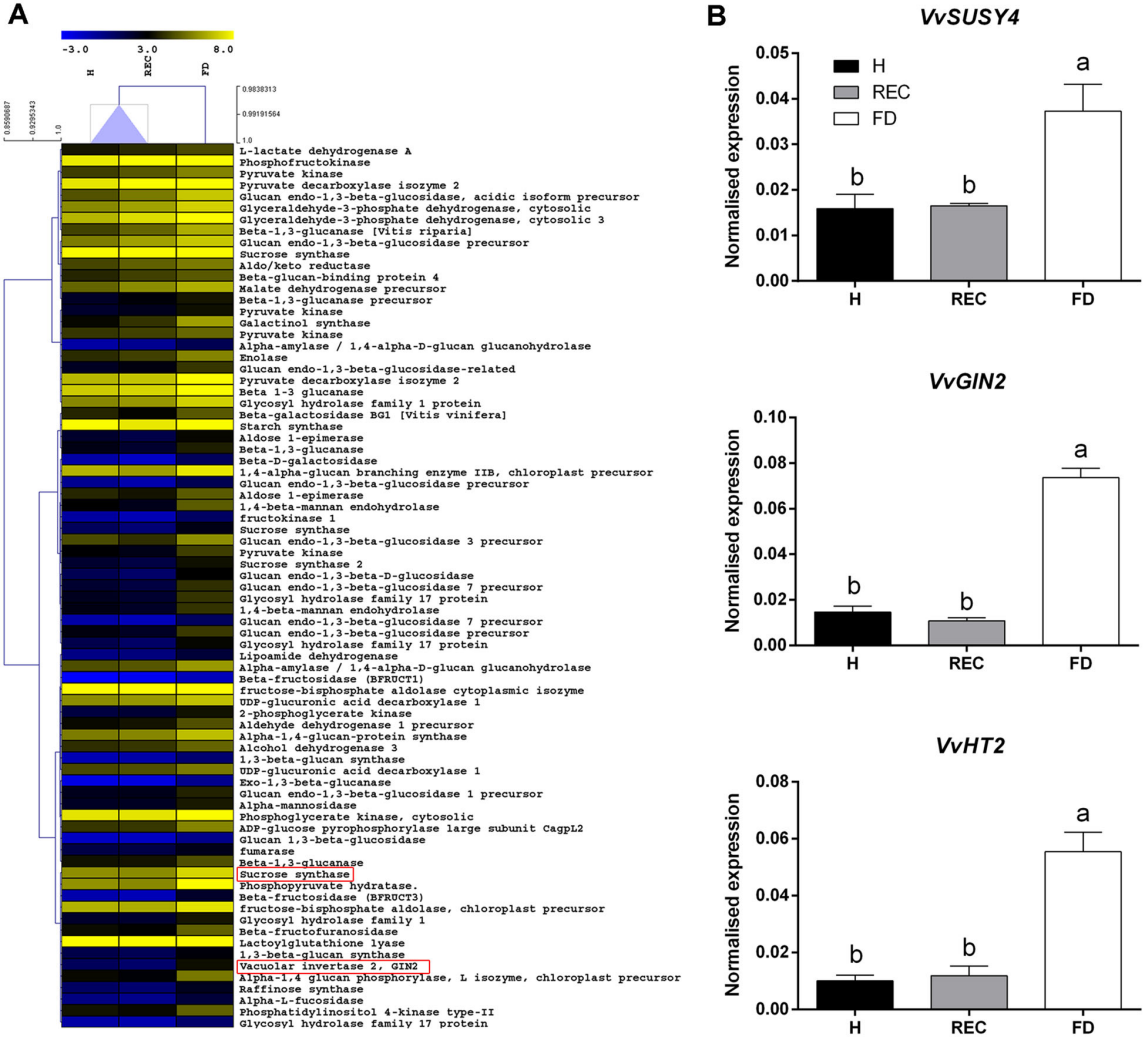
**Effects of phytoplasma infection on carbohydrate metabolic processes.a** Heat map of differentially expressed transcripts belonging to carbohydrate metabolism. **b** Focus on genes encoding key sugar-mobilising enzymes: the sucrose synthase SUSY4 (VIT_11s0016g00470), the vacuolar invertase GIN2 (VIT_02s0154g00090) and the hexose transporter HT2 (VIT_18s0001g05570). RT-qPCR analysis was performed on leaf vein samples from healthy (H), recovered (REC) or FDp-infected (FD) ‘Barbera’ plants. Ubiquitin (*VvUBI*) and actin (*VvACT1*) genes were both used as endogenous controls for the normalisation of transcriptional levels. Lower case letters denote statistical differences when significant, as determined by Tukey’s HSD test (*P* < 0.05). Bars represent standard error of the mean (*n* = 5)

Other FD-dependent clusters enclosed transcripts belonging to carbohydrate metabolism and transport (Fig. 3a), such as the hexose transporter *VvHT2* (VIT_18s0001g05570), the sucrose synthase *VvSUSY4* (VIT_11s0016g00470) and the vacuolar invertase *VvGIN2* (VIT_02s0154g00090). All of them were significantly overexpressed in FD veins (Fig. 3b), following transcriptional patterns close to anthocyanin biosynthetic genes.

These observations are consistent with the role of sugars as signals promoting anthocyanin biosynthesis^39^ and with the activation of these genes in response to pathogen pressure^40^. An exception to this trend was represented by some β1-3 glucanase-encoding transcripts, including *Vvβ1-3gluc* (VIT_08s0007g06040; Supplementary Fig. S4A), which were highly transcribed in both FD and REC samples (Fig. 3a).

**Fig. 4:**
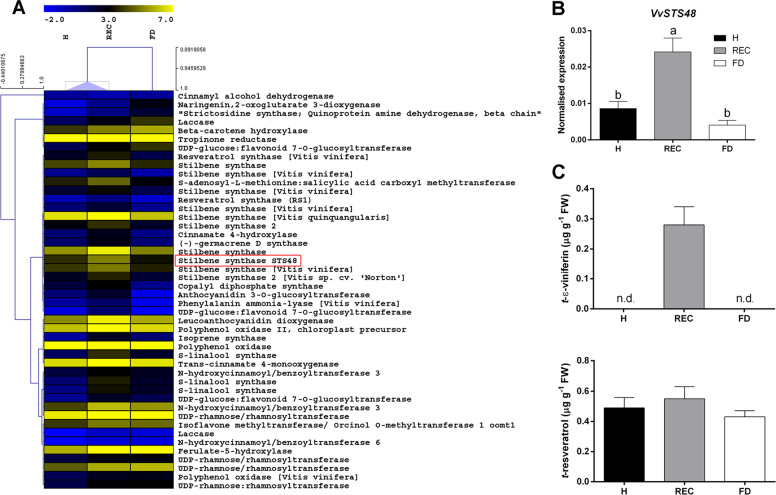
Recovery-dependent modulation of specific secondary metabolic genes. **a** Heat map of the secondary metabolism-associated transcripts overexpressed during recovery. **b** RT-qPCR expression rates of the stilbene synthase gene *VvSTS48* (VIT_16s0100g01200) in ‘Barbera’ leaf veins from healthy (H), recovered (REC) or FDp-infected (FD) plants and **c** accumulation patterns of viniferin and resveratrol analysed in the same samples. Lower case letters denote statistical differences when significant, as determined by Tukey’s HSD test (*P* < 0.05). Bars represent standard error of the mean (*n* = 5). n.d. not detectable

Other phenylpropanoid biosynthetic pathways, such as those leading to stilbene production, were exclusively upregulated by REC (Fig. 4a). Correspondingly, RT-qPCR analysis showed that stilbene synthase (STS) encoding genes, such as *VvSTS48* (VIT_16s0100g01200), were significantly induced in REC samples (Fig. 4b).

Although *STS* transcripts were overall more abundant in REC than FD samples, the comparison between REC and H transcriptomes showed that only some *STS* were upregulated by REC (i.e. *VvSTS48*), whereas others, such as *VvSTS16/22* (VIT_16s0100g00840/VIT_16s0100g00920), had similar expression levels in healthy and recovered vines (Supplementary Fig. S4B).

**Fig. 5:**
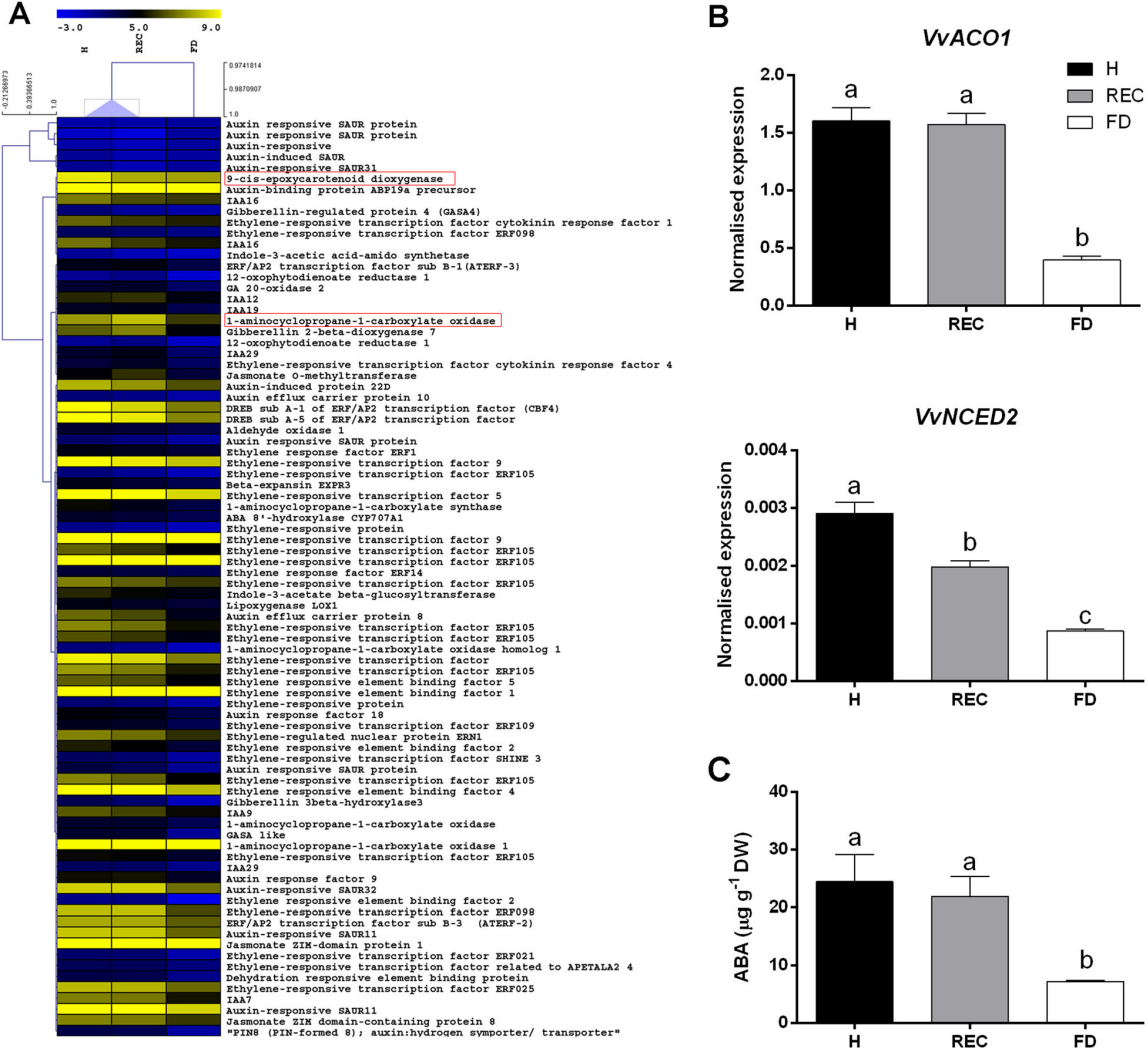
The key hormone metabolic changes associated with recovery and/or healthy status. **a** Heat map of the main differentially expressed transcripts belonging to hormone metabolism and signalling. **b** RT-qPCR expression rates of the ethylene biosynthetic gene *VvACO1* (VIT_00s2086g00010) and of the ABA biosynthetic gene *VvNCED2* (VIT_02s0087g00930) in ‘Barbera’ leaf veins from healthy (H), recovered (REC) or FDp-infected (FD) plants. **c** Patterns of ABA accumulation in the same samples. Lower case letters denote statistical differences when significant, as determined by Tukey’s HSD test (*P* < 0.05). Bars represent standard error of the mean (*n* = 5)

Hormone signalling processes were also differently regulated following FDp infection or recovery. Several transcripts involved in ethylene- and auxin-based signalling pathways were exclusively activated in REC and H veins and clustered separately from FD (Fig. 5a).

Similarly, transcripts linked to abscisic acid (ABA) and jasmonic acid (JA) metabolic processes, including epoxycarotenoid dioxygenases (*NCED*), lipoxygenases and oxophytodienoate reductases, were also negatively modulated by FD condition (Fig. 5a). RT-qPCR performed on the biosynthetic genes 1-aminocyclopropane-1 carboxylase oxidase (*VvACO1*-VIT_00s2086g00010) and 9-*cis*-epoxycarotenoid dioxygenase (*VvNCED2*- (VIT_02s0087g00930)), respectively involved in ethylene and ABA metabolism, confirmed these transcriptional changes (Fig. 5b).

### FD- or REC-induced accumulation of abscisic acid and secondary metabolites

RNAseq data revealed that transcriptional activation of secondary biosynthetic pathways belonging to the phenylpropanoid category was differently regulated by either FD (Fig. 2a) or REC (Fig. 4a). This result was confirmed by the measurement of different molecules, especially of plant defence compounds. Besides the increase in anthocyanins already attested in FDp-diseased ‘Barbera’ plants^7^, monomeric flavan 3-ols, such as catechines, were also more abundant in response to FDp than during recovery. Conversely, cumarins, cinnamates and hydroxycinnamiltartaric acids (HCA), such as caftaric and fertaric acids, and some flavonols, such as quercetin-3-glucuronide and kaempferol-3-glucuronide, were more concentrated in REC and H than in infected veins (Supplementary Table S5), supporting recently published findings^41^. Total stilbenoids, particularly the piceid forms, were instead higher in FD than REC and H veins (Supplementary Table S5). This was unexpected, given the significantly higher transcription of *STS* genes in REC and H than FD samples. Nevertheless, other phytoalexins, such as trans-*ε*-viniferin, were exclusively accumulated in REC samples (Fig. 4c), consistently with REC-dependent activation of specific *STS* genes (Fig. 4b).

As genes belonging to abscisic acid (ABA) metabolism were negatively modulated by FDp (Fig. 5), and a role for this hormone in the regulation of stilbene biosynthesis was proposed^31^, ABA amounts were determined in target samples. Results evidenced that endogenous ABA levels were significantly higher in REC and H than in FD veins (Fig. 5c), in accordance with the expression profile of the ABA biosynthetic gene *VvNCED2* (Fig. 5b) and sharing the same accumulation trend of JA previously reported for the same samples^35^.

### Landscapes of DNA methylation differ in REC and H plants

Comparison of REC and H transcriptomes revealed genes whose expression was apparently modified by long-lasting effects of the FD infection priming event. As this priming took place more than 2 years before we analysed the transcriptome of REC plants (i.e. symptom remission was first observed the year after the original infection, then recovery was diagnostically confirmed for the two following years; thus we referred to as 2-year-old stably recovered vines), we searched for possible epigenetic marks induced by the previous infection and that could underlie stress memory events at the level of DNA methylation status.

**Fig. 6:**
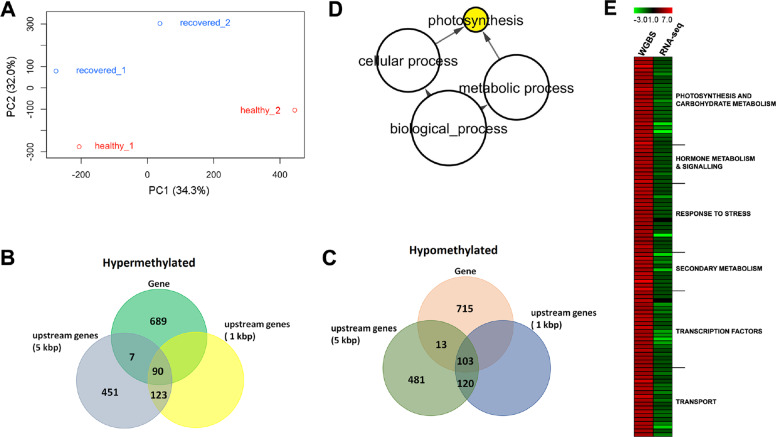
Outcome of WGBS methylome analysis. **a** Plot of principal component analysis displaying the distinction between REC and H methylomes. **b**, **c** Venn diagrams showing the number of hyper- (**b**) and hypomethylated (**c**) genes interested by CpG differential methylation at the level of gene body, 1 and 5 kb upstream regions of REC genes. **d** Enrichment of GO biological process categories for the group of 288 genes CpG hypermethylated at the promoter level matching the RNAseq data (Supplementary Table S10). The significant enriched GO biological processes terms were identified using Cytoscape with the BINGO plug-in and listed according to their enrichment *P*‐value (*P* < 0.05). **e** Merger between WGBS and RNAseq data: the heat map highlights the differential methylation levels of a group of genes CpG hypermethylated at the promoter level (left) in comparison with the downregulation of the corresponding transcripts (right)

To test this hypothesis, we analysed DNA cytosine methylation profiles of REC and H plants by whole-genome bisulfite sequencing (WGBS) approach. Two libraries for each condition were produced with an average final sequencing coverage of 38.4× and an average cytosine conversion efficiency higher than 99.5% (Table S6). A PCA analysis conducted on WGBS data attested that REC and H samples significantly differed in terms of CpG methylation patterns, forming two distinct clusters (Fig. 6a).

A total of 1360 genes were found CpG hypermethylated in REC; for 451 of those DNA methylation marks fell in the 5 kb upstream gene region, whereas for other 123 genes DNA changes also interested the 1 kb upstream gene region. However, the majority of CpG hypermethylated genes (689) underwent modifications occurring within the gene body (Fig. 6b; Supplementary Table S7). CpG hypomethylated genes were slightly more (1432 in total), and also in this case most of the DNA modifications interested either the 5 kb region upstream the gene (481) or the gene sequence itself (715) (Fig. 6c).

GO enrichment analysis carried out on hyper- and hypomethylated genes, either considering them separately or together, did not highlight the presence of significant over-represented functional gene categories. Nevertheless, when methylome results were merged with RNAseq data deriving from the REC vs H comparison, a correspondence between CpG methylation levels and transcriptional rates was highlighted in 610 out of the total of hypermethylated genes and in 616 out of the total of hypomethylated genes (Supplementary Table S8). A new GO enrichment analysis run on the subset of 288 genes CpG hypermethylated at the promoter level and matching the RNAseq data (Supplementary Table S9) showed significant overrepresentation of functional classes associated with Photosynthesis (Fig. 6d), that were downregulated by FD. This group of hypermethylated genes in REC plants also included transcripts belonging to transport and secondary metabolic processes that were previously detected among the genes mostly upregulated by FD (e.g. *HT2*, *AOMT*, *CHS3*) and that in parallel were inhibited by recovery and/or healthy status (Supplementary Table S9; Fig. 6e).

## Discussion

### Global molecular and metabolic modifications triggered by FD

In order to search for molecular changes induced by *Flavescence dorée* in leaves of the highly susceptible Barbera cultivar^15^, the transcriptomes of diseased, stably recovered (2-year-old recovery) and healthy vein enriched-samples were analysed.

Global transcriptomic changes occurring in FDp-infected plants confirmed previous observations linking FD symptomatology to severe photosynthetic limitations^14^ and increased accumulation of soluble sugars^32^ and flavonoids in the leaf^7^. The carbohydrate metabolic category was one of the most affected by the disease. Phytoplasmas are completely dependent on the host metabolism for sustaining their multiplication^42^. Infection by these phloem-restricted pathogens induces sugar mobilisation (e.g. glucose) in the leaf through increased activity of sucrose synthases and vacuolar invertases^32^. Consistently, transcripts encoding these enzymes (*SUSY4* and *GIN2*, respectively) were exclusively overexpressed under FDp infection, as well as genes involved in hexose transport (*HT2*) and starch metabolism (*AGPase* and *alpha-amylase*). In addition, these transcriptional changes agreed with the expression patterns of carbohydrate biosynthetic and transport-related genes reported in BNp-infected grapevines^11,18^. Sugars are crucial stress-signalling molecules involved in a wide number of physiological processes^39^. It is thus conceivable that the FD-mediated induction of genes encoding sugar-mobilising enzymes triggers the onset of defence responses to limit the pathogen spread. These responses may include anomalous callose deposition in the sieve elements of infected plants^10^ associated with impaired phloem loading^10,11,32^ that in turn slows down the Calvin cycle^17^. The downregulation of photosystem I and II (*PSI*, *PSBx*) and of chlorophyll biosynthetic (*CHL*) genes, here observed, is consistent with this hypothesis. In addition, FD-induced sugar accumulation could drive the overexpression of anthocyanin biosynthetic genes (i.e. *UFGT*, *AOMT*, *3AT*), therefore supporting the intense leaf reddening phenotype of FDp-infected ‘Barbera’ plants. As suggested previously^7^, the high recovery aptitude of ‘Barbera’ to spontaneous symptom remission^13,15^ may imply that this severe mobilisation of sugars and antioxidant compounds is a particularly effective strategy for counteracting the pathogen spread and preserving the plant tissues from further oxidative damage.

### Specific molecular and metabolic changes underly the establishment of recovery

Some FD-induced transcriptional modifications persist under recovery, such as those involving photosynthetic genes, as evidenced by comparing FD and REC samples. Nevertheless, when REC and H transcriptomes were compared a few total significant DEGs were found, revealing that REC ‘Barbera’ plants are similar to the healthy ones at the transcriptomic level, in line with their symptomless phenotype. Particularly, transcriptional changes underlying recovery are associated with a complete reprogramming of stress-mediated responses previously triggered by the infection and mainly affecting secondary and hormone metabolisms. Accordingly, hydroxycinnamates, the predominant group of non-flavonoid phenols in grape^43^, are specifically accumulated in REC samples (Supplementary Table S5), suggesting that the reduced amount of anthocyanins in the leaf veins of REC plants may divert carbon to the biosynthesis of phenylpropanoids^41^.

The high concentrations of total stilbenoids in diseased samples supports the strong defence responses of infected ‘Barbera’ plants to counteract the disease. However, in REC veins, the overexpression of many stilbene synthase-encoding genes is exclusive, as well as the presence of specific categories of stilbenoids, such as viniferin, known to have a pivotal antimicrobial role in grapevine^23^.

Another distinct feature of REC transcriptome is the upregulation of hormonal metabolic pathways associated with ABA, JA, auxin and ethylene. Accumulating experimental evidence has supported a role for these hormones in plant immunity^30^, and previous target expression analyses revealed a significant overexpression of transcripts linked to their metabolic and signalling pathways in recovered grapevines^16,44^. Experiments conducted in cultivated grapevines or grape cell cultures demonstrated that treatment with either exogenous JA or ABA can regulate the production of stilbenes, in particular by stimulating the accumulation of viniferin^31^. It is thus feasible that the transcriptional activation of these hormonal signalling pathways, consistently with the observed concentration patterns of ABA (this study) and JA^35^, may facilitate the establishment of recovery by triggering the synthesis of antimicrobial compounds.

Furthermore, the merger between the RNAseq (this study) and smallRNAseq data sets^35^ highlighted a novel network of regulatory signalling cascades involved in the recovery process. Several genes involved in ABA and JA metabolism are targeted by miRNA activity. For instance, the interplay among TEOSINTE BRANCHED/CYCLOIDEA/PCF (*VvTCP*, VIT_12s0028g02520)/vvi-miR319, JA ZIM domain-containing protein (*VvJAZ3*, VIT_01s0011g05560) targeted by vvi-miR169, vvi_miC197-5p and vvi_miC52-5p, and 12-oxophytodienoate reductase (*VvOPR3*, VIT_01s0011g05560)/vvi_miC481-5p finely tunes JA signalling cascades^35^. Interestingly, the REC-dependent activation of the JA pathway could serve to achieve and maintain the recovery status by inhibiting the SA-signalling, which is rather associated to defence responses following pathogen infection^9,30^.

### Multiple molecular players contribute to recovery following FDp infection

It was recently advanced that differences in CpG methylation profiles can facilitate the rapid adaptation of plants to changing environmental conditions^45,46^ as well as responses to pathogens^20^. Epigenetic marks underlying defence mechanisms in grapevine are still hotly debated^21^ and to our knowledge, there are no studies exploring naturally occurring patterns of DNA methylation associated to recovery from phytoplasma infection in field-grown plants. Indeed, available information takes into account either effect of artificially induced DNA methylation in phytoplasma-infected plants maintained under controlled greenhouse conditions^47^ or histone modifications occurring in in vitro-cultured phytoplasma-diseased plantlets^48^. Changes in DNA methylation patterns were analysed only on in vitro periwinkle plants exposed to exogenous auxin treatment, in order to investigate effects of hormonal treatments on recovery induction^49^. Finally, Osler et al.^50^ reported that apricots obtained by grafting-mediated propagation of European stone fruit yellows (ESFY)-recovered plants were less prone to new ESFY infection, thus hypothesising graft-transmissible stress memory signals promoting acquired tolerance phenomena in the offspring. However, unlike ESFY-recovered plants, FD recovered vines do not retain the phytoplasma and returned negative to PCR diagnostic assay^8,15^. Although it cannot be excluded that FDp could persist in recovered vines at concentrations below the detectable PCR threshold, a multi-year study performed in vineyards of north-western Italy, including the one used in our experiment, evidences that the vector insect is not able to acquire the FD phytoplasma by feeding on recovered vines, and that it does exist a significant positive correlation between the vector acquisition efficiency and FDp titre in the analysed plants^51^. The inability of recovered plants to serve as source of inoculum for the vector may thus imply the absence of FDp in the phloem tissues. This is consistent with long-standing observations achieved by our research team working on potted vines artificially infected with the vector and maintained in dedicated screen houses equipped with nets preserving from insects: once recovered, the plants have no longer shown FD symptoms and remained PCR negative for the following years (data not shown).

Another possibility is that, out of the summer time-frame typically suited for FD diagnosis, FDp could persist in recovered plants within tissues such as roots, in which, although at low concentration, it could remain active keeping on alert the defence machinery of those vines. For instance, in the case of Bois Noir disease, the pathogen presence was successfully detected in roots from field-grown infected and recovered vines^52^. However, the low number of root samples found positive to the PCR diagnostic tests, in combination with the much lower BNp titres quantified in recovered than infected plants, leave open the possibility that over time the phytoplasma might disappear in grapevine roots^52^. Notably, there is no literature attesting the FDp presence in roots (or tissues other than leaves or shoots) of *V. vinifera* cultivars. Accordingly, in a separate experiment, we screened roots collected from infected, recovered and healthy potted ‘Barbera’ plants during the vegetative and winter seasons, and we were not able to detect presence of the phytoplasma using the same PCR diagnostic method described in this study (data not shown). Collectively, this information strongly suggests that biological mechanisms at the basis of FD recovery should be searched in intrinsic defence process previously primed in the host during the infection^12,16^. Accordingly, Pacifico et al.^44^ showed significant differences in the transcriptional profiles of defence-associated genes when leaves from healthy and 2-year-old-recovered plants of ‘Barbera’ and ‘Nebbiolo’ were analysed, consequently hypothesising stress-induced long-lasting effects.

In order to assess whether recovery may be induced by the priming effect of FDp infection, we explored the landscapes of DNA methylation at CpG sites in REC plants, and we compared them with transcriptomic changes observed in FD vs H and REC vs H plants. The different methylation profiles of REC and H samples highlight a new level of regulation of the recovery process. Recovery from FDp infection corresponds with a general switching off genes involved in carbohydrate metabolism (e.g. *VvSUSY4*, *VvGIN2*), flavonoid metabolism (e.g. *VvCHS3*, *VvUFGT*, *Vv3AT*, *VvAOMT*) and anthocyanin and sugar mobilisation (e.g. *VvGST4* and *VvHT2*, respectively), all belonging to key metabolic nodes positively affected by FD. Several genes that are highly expressed in FDp-infected plants are hypermethylated in REC, in accordance with the corresponding downregulation patterns found in the FD vs REC and REC vs H transcriptome comparisons. This indicates that DNA methylation analysis may help discriminating H from REC grapevines and overcoming the effects of climatic and edaphic conditions on a single time-point transcriptomic snapshot^44^.

Another interesting result here is that genes belonging to key downregulated metabolic processes in FD and REC, such as photosynthesis-associated genes, undergo multiple levels of regulation, being the substrate of post-transcriptional control mediated by small RNAs^35^ and of cytosine DNA methylation changes (this study). Transcripts encoding the Photosystem I reaction centre subunit II and Photosystem II subunit X, both downregulated in FD and REC samples, are respectively targeted by the conserved vvi_miR169 and the novel vvi_miC137-3p^35^. The CpG hypermethylation detected at the promoter level of some photosynthetic genes in the REC genome may also contribute to lower photosynthesis-related transcriptional rates. This synergy between transcriptional and post-transcriptional regulatory pathways could ultimately lead to decrease photosynthetic efficiency of REC plants^14^. Hence, changes in methylation landscapes of REC and H genomes may represent a reminiscence of the previous infection that would promote molecular memory phenomena controlling the recovery maintenance in previously FDp-infected grapevines.

In plant species commonly multiplied through sexual reproduction, the transgenerational transmission of epigenetic marks might allow for the rapid adaptation of plant populations to changing environmental cues^53^. Nevertheless, in perennial vegetative multiplied plants, mechanisms behind the transgenerational maintenance of epigenetic variation are still largely unknown^54^. Excluding model plants^46^, experimental evidence on stress-induced molecular memory is hardly limited in crop species^55^, and a clear understanding of the maintenance of a plant’s primed state facilitating the establishment of long-lasting molecular memory effects has not been achieved yet^56^. Therefore, especially in the case of clonal plant populations growing in open field conditions^57^, like grapevine, the study of stress recovery phenomena could provide useful information to dissect when, how, and why molecular memory events are established.

## Conclusion

To our knowledge, this is the first application of integrated high throughput sequencing technologies for providing a comprehensive overview of transcriptional changes caused by the grapevine *Flavescence dorée* phytoplasma, and for gaining novel insights into the understanding of spontaneous recovery from the disease. The comparison among the transcriptomes of FD, REC and H vein enriched-leaf samples, supported by methylome analysis and by quantification of hormones and polyphenols, allowed us to deepen stress-mediated molecular responses previously suggested by candidate gene expression analyses, and to elucidate molecular regulatory steps affecting the primary and secondary metabolic changes underlying recovery.

We pointed out that the recovery status associated to symptomless phenotypes is maintained through transcriptional reprogramming processes, switching off biochemical signals previously triggered by the pathogen, and through DNA methylation events. It thus emerged that stably recovered grapevines still harbour signs of phytoplasma infection that may result from the establishment of molecular memory phenomena.

## Materials and methods

### Plant material and experimental outline

The trial was conducted in summer 2014 on plants of the red-grape *V. vinifera* cultivar Barbera grown in a productive vineyard implanted in 1999 (Cocconato (AT), gps data: 45°04′58.4″N 8°03′21.1″E, N-S orientation, 350 m a.s.l.), in which single-plant events of phytoplasma infection had been constantly monitored since 2007^7^. The vineyard (1.75 ha) consisted of 76 rows of vines, with a density of 5000 plants ha^−1^, grafted onto the SO4 rootstock. As a preliminary step of the study, the presence of the main phytoplasmas (FDp and BNp) affecting European grapevines was inspected on target plants at three time points during the growing season (late June, late July and late August), using the protocol by^58^. Plants were also checked for the nine most important grapevine viruses in Italy [arabis mosaic virus (ArMV), grapevine fanleaf virus (GFLV), grapevine virus B (GVB), grapevine fleck virus (GFkV), grapevine leafroll-associated virus-1, -2, -3 (GLRaV-1, -2, -3), grapevine virus A (GVA) and grapevine rupestris stem pitting-associated virus (GRSPaV)] according to^59^. Only plants free of the tested viruses and of BNp were chosen for sample collection. The experimental categories, each constituted by five vines of the same age and located on neighbouring rows, were as follows: FDp-infected (FD), stably recovered (REC, i.e. plants found positive to FDp in the past, but then shown to be FDp-negative and symptomless for at least two consecutive years) and healthy (H). For REC and H plants, leaves were randomly collected from the central region of the shoot, whereas leaves from FD vines were taken paying attention to the presence of the typical FD symptoms^4^. Samples collected in late July, the moment of maximum infection according to the molecular diagnosis data^15^, were used for molecular and biochemical analyses. Veins were isolated from whole leaf tissues using a sterile scalpel, immediately frozen in liquid nitrogen, and stored at −80 °C until use. The five plants selected for each sanitary condition constituted independent biological replicates. Three of them were used as independent biological replicates for RNAseq and metabolic analyses, whereas all five plants were subjected to real-time PCR assays.

### RNA library preparation, sequencing and bioinformatic analyses

Total RNA was extracted in triplicate from previously isolated leaf veins using the Spectrum™ Plant Total RNA extraction kit (Sigma-Aldrich, Inc.). Quantity and quality of RNA samples were checked by Bioanalyzer assay (2100 Bioanalyzer, Agilent Technologies), and only samples showing RIN (RNA integrity number) > 8 were used for library preparation and for real-time PCR analyses. Nine (three per condition) cDNA libraries were prepared using the TruSeq RNA Sample Prep Kit v. 2 (Illumina, San Diego, USA) according to manufacturer’s instructions, and sequenced using the NextSeq500 system platform (Illumina, San Diego, USA) by an external service (Functional Genomics Lab, University of Verona). Pre-processing of the data, alignment of the reads, and identification of differentially expressed genes (DEGs) were performed as previously described^60^. Transcripts were annotated using the V1 version of the 12× draft annotation of the grapevine genome (http://genomes.cribi.unipd.it/DATA/). The GO biological process classification and VitisNet GO annotations^61^ were adopted for grouping into functional gene classes (Supplementary Table S2). Hierarchical clustering (HCL) analysis was run using Pearson’s correlation distance and the MeV software (v.4.9, http://www.tm4.org/mev.html), using as input log2 transformed FPKM values.

GO enrichment analysis was carried out using the BiNGO 3.0 plug-in tool in Cytoscape v. 3.2^62^, and over-represented Plant GO slim categories were identified using a hypergeometric test with a significance threshold of 0.05.

### Whole-genome bisulfite sequencing of DNA (WGBS) and data elaboration

Genomic DNA was extracted according to^63^ starting from leaf veins collected from two REC and two H vines among the plants selected for RNAseq and metabolic analyses (see above). The four WGBS libraries were constructed using the Illumina TruSeq DNA Methylation Kit after processing with EZ DNA Methylation‐Gold™ kit (Zymo Research, https://www.zymoresearch.com), and sequenced by an external service (Institute of Applied Genomics, Udine), using the NovaSeq 6000 sequencing system yielding ~300 million paired-end 150 bp reads per library.

Bisulfite-treated read mapping and methylation calling were carried out using Bismark v0.20.0^64^ and the PN40024 genome v2^65^ with gene annotation v2.1^66^ as reference. Prior to mapping, bisulfite-treated reads were trimmed with TrimGalore v0.5 (Cutadpt v1.15; --length 80) and then randomly subsampled to 81,941,149 reads/library to obtain an even number of reads across replicates and treatments. Bisulfite conversion efficiency was estimated based on conversion of the unmethylated lambda-phage DNA spiked-in prior to bisulfite treatment. Conversion efficiency was greater than 0.995 for all samples. Methylation calls were analysed using methylKit v1.2.4^67^. Base calls with coverage below 10× or more than 99.9th percentile in each sample were discarded (filterByCoverage with parameters lo.count=10,hi.perc=99.9). Methylation counts were calculated over 1 kb tiling windows (tileMethylCounts with parameters win.size=1000, step.size=500). Differential methylation was computed using the getMethylDiff function with *q*-value < 0.01 and percent methylation difference > 25%.

### Real-time PCR analysis

First strand cDNA was synthesised starting from 500 ng of total RNA previously treated with DNase I (Invitrogen, ThermoFisher Scientific) using the High Capacity cDNA Reverse Transcription kit (Applied Biosystems, ThermoFisher Scientific) and following manufacturer’s instructions. Real-time PCR reactions were performed according to^16^. Ubiquitin (*VvUBI*) and Actin (*VvACT1*) reference genes were used as internal controls for normalising transcript expression levels. Oligonucleotides used in real-time PCR experiments are listed in Supplementary Table S10. Five biological replicates per treatment were analysed and three technical replicates were run for each of them.

### Analysis of secondary metabolites

Flavonoids and stilbenes were identified and quantified on the samples used for RNAseq by Ultra High Performance Liquid Chromatography (UHPLC), according to^68^. Samples were prepared as described in^69^. Briefly, 0.4 mL of chloroform and 0.6 mL of methanol:water (2:1 v/v) were added to 0.1 g of fresh sample. The extraction was repeated by adding 0.6 mL of methanol and water (2:1 v/v) and 0.2 mL of chloroform. The aqueous-methanolic phases were combined and evaporated to dryness under N_2_. Samples were re-suspended in 500 µL of methanol and water (1:1 v/v), centrifuged and transferred into an HPLC vial. Chromatographic analysis was performed using a Waters Acquity UPLC system (Milford) equipped with a Waters Acquity HSS T3 column using the solvents B (acetonitrile containing 0.1% formic acid) and A (water containing 0.1% formic acid). Mass spectrometry detection was performed on a Waters Xevo triple-quadrupole mass spectrometer detector (Milford) with an electrospray (ESI) source^68^. Compounds were identified based on their reference standards, retention time and qualifier and quantifier ion, and were quantified using their calibration curves. Data were finally expressed as µg g^−1^ of fresh weight (FW). Data processing was performed using the Waters MassLynx V4.1 software.

### Analysis of abscisic acid (ABA) content

Leaf veins (40 mg) were freeze dried and homogenised, then transferred in a 2 mL centrifuge tube and extracted with 1 mL of methanol: water (1:1 v/v) acidified with 0.1% of formic acid in an ultrasonic bath for 1 h. Samples were centrifuged at 15,000 rpm and 4 °C for 10 min, and the supernatant was analysed by HPLC-DAD technique. Original standard of ABA (purity ≥ 98.5%; Sigma-Aldrich) was used for metabolite identification by comparing retention times and UV spectra. Metabolite quantification was made by external calibration method. An Agilent 1220 Infinity LC system model G4290B (Agilent®, Waldbronn, Germany), equipped with gradient pump, autosampler and column oven set at 30 °C, and a 170 Diode Array Detector (Gilson, Middleton, The USA) set at 265 nm were employed. A Nucleodur C18 analytical column (250 × 4.6 mm i.d., 5 μm, Macherey Nagel) was used. The mobile phases consisted of water acidified with formic acid 0.1% (A) and acetonitrile (B), at a flow rate of 0.600 mL min^−1^ in gradient mode, 0–6 min: 30% of B, 6–16 min: from 30 to 100% B, 16–21 min: 100% B. Twenty μL were injected for each sample and three biological replicates were run for each analysis.

### Statistical analyses

Significant differences among treatments were statistically analysed by one-way ANOVA test, using the Tukey’s HSD post-hoc test for separating means when ANOVA results were significant (*P* < 0.05). Significant differences of pairwise comparisons were assessed by the Student’s *t*-test. The SPSS statistical software package (SPSS Inc., Cary, NC, USA, v.24) and the GraphPad Prism software (GraphPad Software, La Jolla, CA, USA v.6.01) were used to run the statistical analyses above reported and elaborate figure charts, respectively.

## Supplementary information

Supplementary Figure S1

Supplementary Figure S2

Supplementary Figure S3

Supplementary Figure S4

Supplementary Table S1

Supplementary Table S2

Supplementary Table S3

Supplementary Table S4

Supplementary Table S5

Supplementary Table S6

Supplementary Table S7

Supplementary Table S8

Supplementary Table S9

Supplementary Table S10

## Data Availability

Raw sequences from the RNAseq and WGBS libraries were deposited at the NCBI Sequence Read Archive under the project numbers PRJNA587882 and PRJNA580028, respectively.
