# Supplementary material for: Molecular memory of Flavescence dorée phytoplasma in recovering grapevines
Source: Hortic Res. 2020 Aug 1;7:126. doi: 10.1038/s41438-020-00348-3 (PMC7395728; doi:10.1038/s41438-020-00348-3)

**Fig. S1.** Enrichment of GO biological process categories for A) the up-regulated and B) down-regulated DEGs resulting from the FD vs REC transcriptme comparison. The significant enriched GO biological process terms were identified using Cytoscape with the BINGO plug-in and listed according to their enrichment P-value ($P < 0.05$).

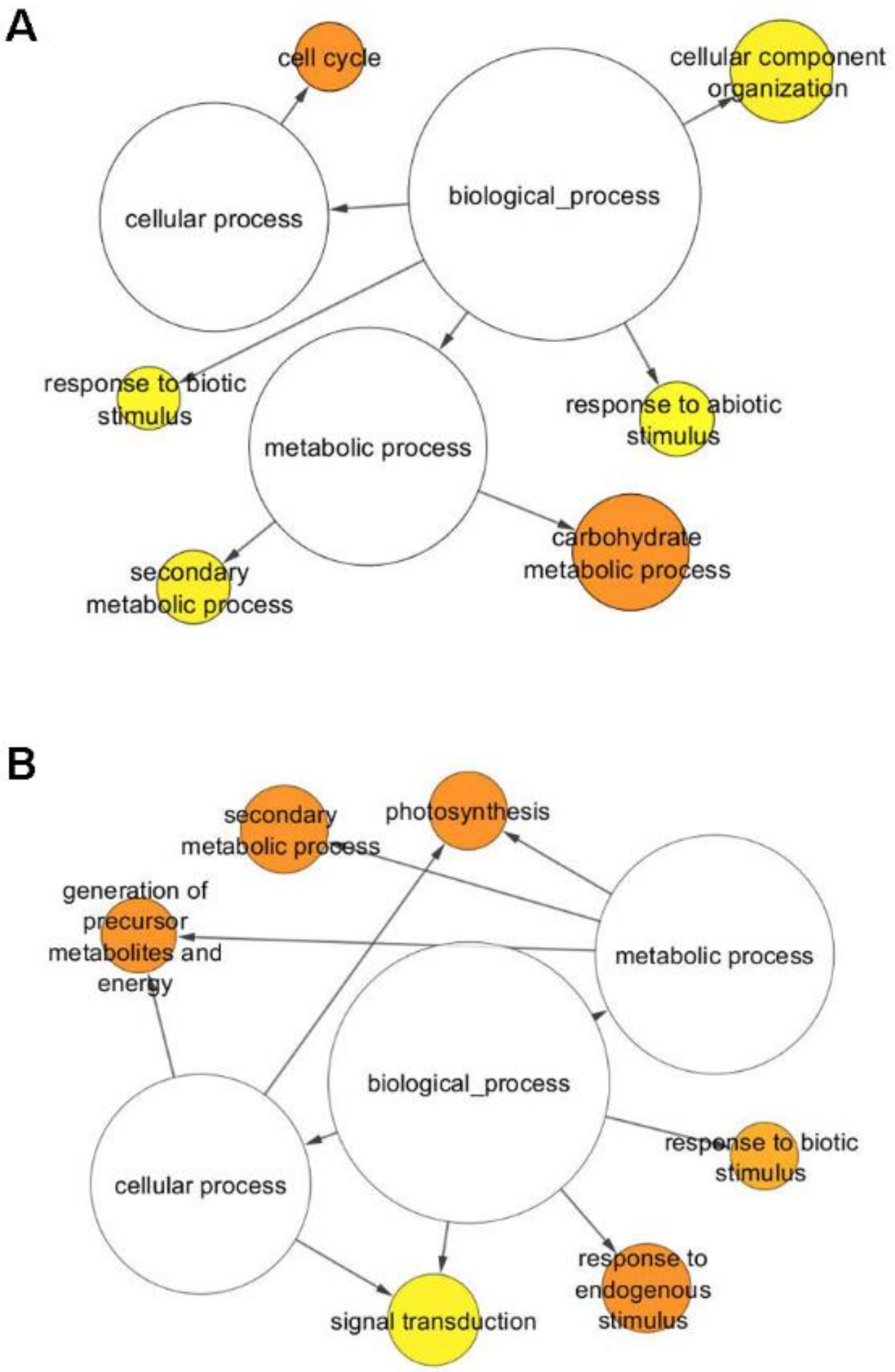

Supplement: Supplementary file 1 — Supplementary Figure S1 [file 41438_2020_348_MOESM1_ESM.pdf]
