# Supplementary material for: Molecular memory of Flavescence dorée phytoplasma in recovering grapevines
Source: Hortic Res. 2020 Aug 1;7:126. doi: 10.1038/s41438-020-00348-3 (PMC7395728; doi:10.1038/s41438-020-00348-3)

Fig. S2. Enrichment of GO biological process categories for the 163 DEGs common to miRNA targets and reported in Table S4. The analysis was performed on the group of miRNA target transcripts (according to[35]) showing significant expression differences in at least one of the three RNA-seq comparisons (FD vs H, REC vs H and FD vs REC; Table S4). The significant enriched GO biological process terms were identified using Cytoscape with the BINGO plug-in and listed according to their enrichment P-value ($P < 0.05$).

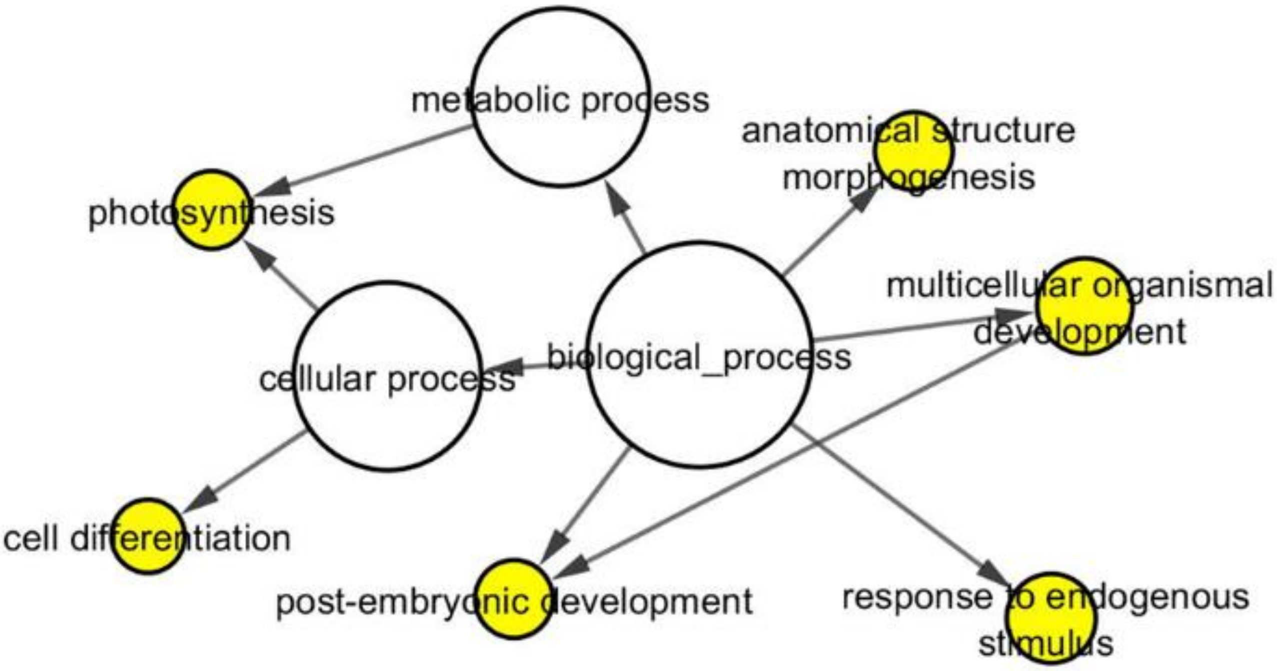

Supplement: Supplementary file 2 — Supplementary Figure S2 [file 41438_2020_348_MOESM2_ESM.pdf]
