# Supplementary material for: Molecular memory of Flavescence dorée phytoplasma in recovering grapevines
Source: Hortic Res. 2020 Aug 1;7:126. doi: 10.1038/s41438-020-00348-3 (PMC7395728; doi:10.1038/s41438-020-00348-3)

Fig. S3. Correlation between RNA seq (FPKM) and RT qPCR results (normalized expression values) obtained from data of the expression analysis performed on transcripts (Table S10) used for validation of RNA seq results. Asterisks mark the statistical significance of the linear regression fit (*P value* < 0.0001).

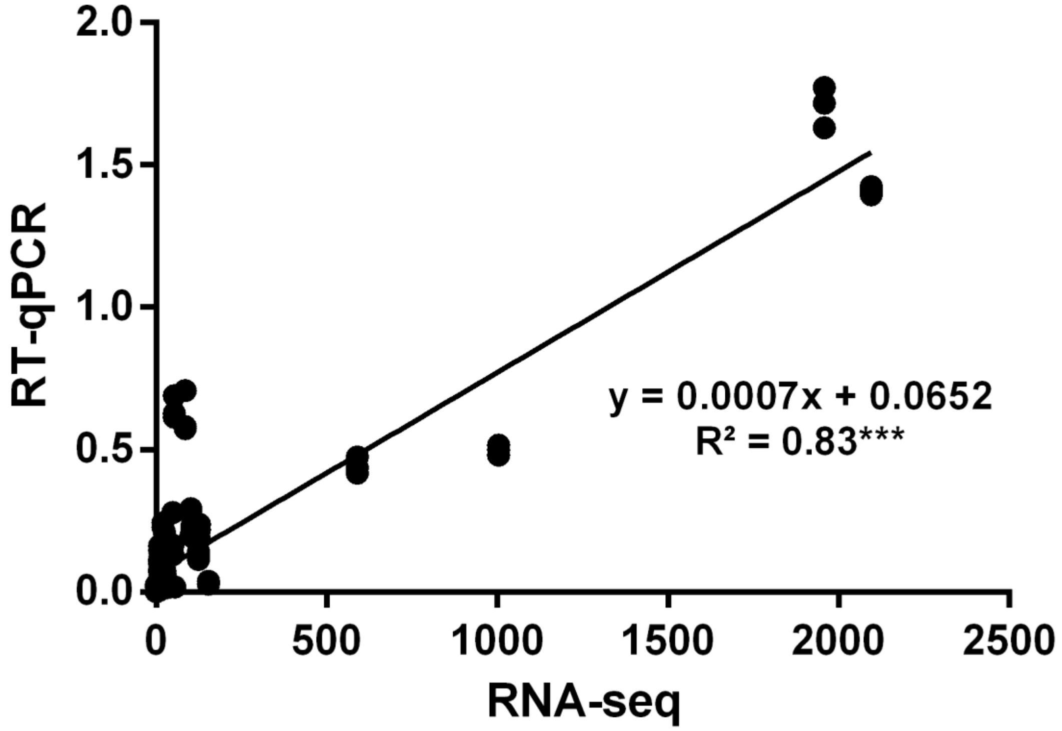

Supplement: Supplementary file 3 — Supplementary Figure S3 [file 41438_2020_348_MOESM3_ESM.pdf]
