# Supplementary material for: Molecular memory of Flavescence dorée phytoplasma in recovering grapevines
Source: Hortic Res. 2020 Aug 1;7:126. doi: 10.1038/s41438-020-00348-3 (PMC7395728; doi:10.1038/s41438-020-00348-3)

Fig. S4. Transcriptional patterns of genes involved in sugar and stilbene metabolism. RT-qPCR expression profiles of *β1-3* glucanase (*Vvβ1-3gluc;* VIT_08s0007g06040) and stilbene synthase (*VvSTS16/22*; VIT_16s0100g00840/VIT_16s0100g00920) genes in leaf veins taken from healthy (H), recovered (REC) or FDp-infected (FD) 'Barbera' plants. Ubiquitin (*VvUBI*) and actin (*VvACT1*) genes were both used as endogenous controls for the normalization of transcriptional levels. Lower case letters denote significant differences as determined by Tukey's *HSD* test ($P<0.05$). Bars represent standard error of the mean ($n = 5$).

A

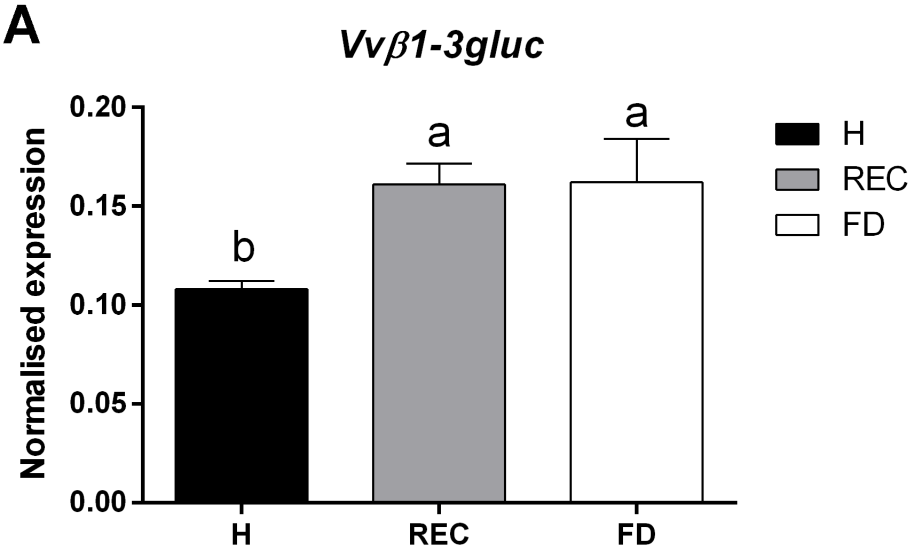

B

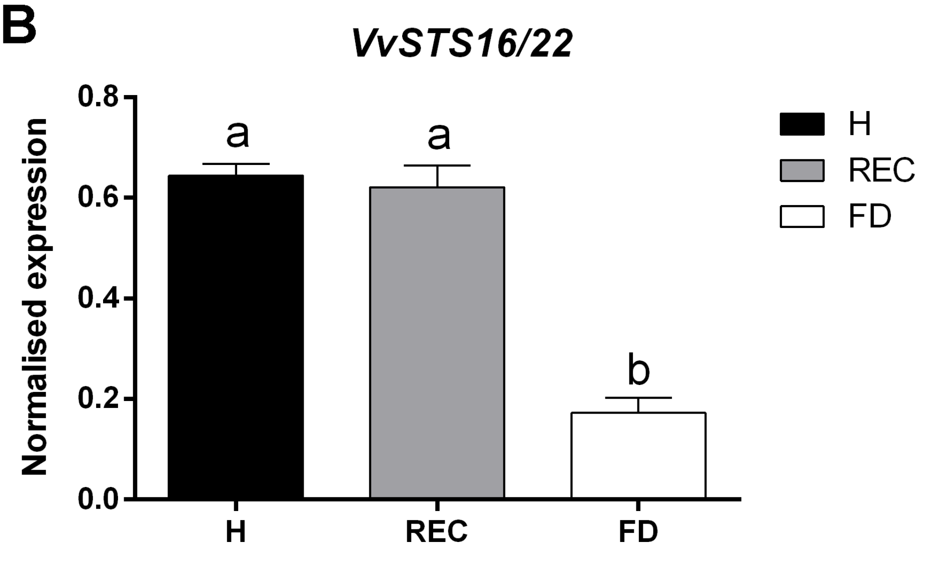

Supplement: Supplementary file 4 — Supplementary Figure S4 [file 41438_2020_348_MOESM4_ESM.pdf]
